# (Isopropyl­amino)(meth­yl)diphenyl­phospho­nium iodide

**DOI:** 10.1107/S1600536811020332

**Published:** 2011-06-18

**Authors:** Normen Peulecke, Stephan Peitz, Bernd H. Müller, Anke Spannenberg, Uwe Rosenthal

**Affiliations:** a Leibniz-Institut für Katalyse e. V. an der Universität Rostock, Albert-Einstein-Strasse 29A, 18059 Rostock, Germany

## Abstract

The title compound, C_16_H_21_NP^+^·I^−^, was obtained by the reaction of Ph_2_PN(^i^Pr)P(Ph)N(^i^Pr)H with MeI involving cleavage of one of the P—N bonds in diethyl ether. The two phenyl rings form a dihedral angle of 82.98 (5)°. A weak donor–acceptor N—H⋯I inter­action is observed.

## Related literature

For the synthesis of Ph_2_PN(^i^Pr)P(Ph)N(^i^Pr)H, see: Peitz *et al.* (2010 ▶). For the structures of amido­phospho­nium salts with similar substituents, see: Payne *et al.* (1965 ▶); Imrie *et al.* (1995 ▶); Aladzheva *et al.* (2003 ▶); Demange *et al.* (2006 ▶); Mizuta *et al.* (2007 ▶).
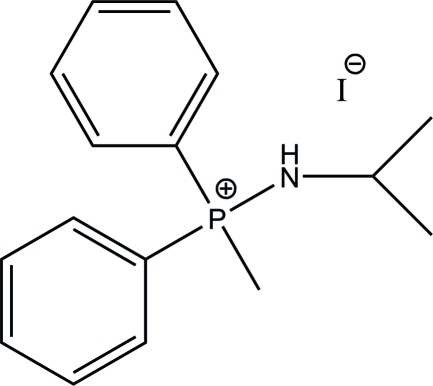


## Experimental

### 

#### Crystal data

C_16_H_21_NP^+^·I^−^
*M* *_r_* = 385.21
Monoclinic, 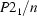

*a* = 9.0283 (2) Å
*b* = 20.2810 (6) Å
*c* = 9.2298 (3) Å
β = 93.492 (2)°
*V* = 1686.87 (8) Å^3^
*Z* = 4
Mo *K*α radiation
μ = 1.98 mm^−1^
*T* = 150 K
0.45 × 0.30 × 0.16 mm

#### Data collection

Stoe IPDS II diffractometer
Absorption correction: numerical (*X-SHAPE* and *X-RED32*; Stoe & Cie, 2005 ▶) *T*_min_ = 0.484, *T*_max_ = 0.741
28937 measured reflections
4035 independent reflections
3604 reflections with *I* > 2σ(*I*)
*R*_int_ = 0.024

#### Refinement

*R*[*F* ^2^ > 2σ(*F* ^2^)] = 0.019
*wR*(*F* ^2^) = 0.048
*S* = 1.05
4035 reflections
179 parameters
H atoms treated by a mixture of independent and constrained refinement
Δρ_max_ = 0.63 e Å^−3^
Δρ_min_ = −0.43 e Å^−3^

### 

Data collection: *X-AREA* (Stoe & Cie, 2005 ▶); cell refinement: *X-AREA*; data reduction: *X-AREA*; program(s) used to solve structure: *SHELXS97* (Sheldrick, 2008 ▶); program(s) used to refine structure: *SHELXL97* (Sheldrick, 2008 ▶); molecular graphics: *SHELXTL* (Sheldrick, 2008 ▶); software used to prepare material for publication: *SHELXTL*.

## Supplementary Material

Crystal structure: contains datablock(s) I, global. DOI: 10.1107/S1600536811020332/ya2139sup1.cif

Structure factors: contains datablock(s) I. DOI: 10.1107/S1600536811020332/ya2139Isup2.hkl

Supplementary material file. DOI: 10.1107/S1600536811020332/ya2139Isup3.cml

Additional supplementary materials:  crystallographic information; 3D view; checkCIF report

## Figures and Tables

**Table 1:** Hydrogen-bond geometry (Å, °)

*D*—H⋯*A*	*D*—H	H⋯*A*	*D*⋯*A*	*D*—H⋯*A*
N1—H1*A*⋯I1	0.81 (2)	2.88 (2)	3.6641 (15)	164.9 (18)

**Fig. 1.**
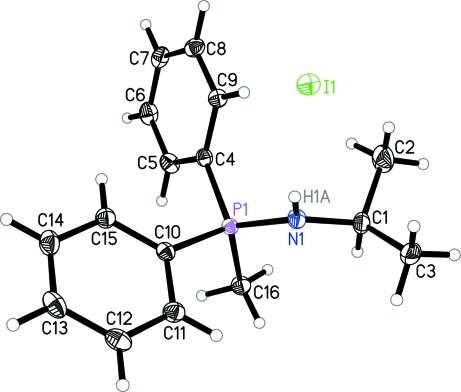
The molecular structure of the title compound; displacement ellipsoids are drawn at the 30% probability level.

## supplementary crystallographic information

### Comment

Amidophosphonium salts are useful precursors for phosphazenes, which are necessary for the aza-Wittig reaction. They can be synthesized by different methods: addition of alkylhalides or methyltriflate to a phosphorus-nitrogen-single bond (Payne *et al.*, 1965; Mizuta *et al.*, 2007), addition of alkylhalides to a phosphorus-nitrogen double bond (Imrie *et al.*, 1995), *via* intramolecular addition of alkylhalides (Aladzheva *et al.*, 2003) or *via* other routes (Demange *et al.*, 2006). We became interested in this class of compounds, because the starting compound Ph_2_PN(^i^Pr)P(Ph)N(^i^Pr)H acts as a superb ligand in the chromium catalyzed trimerization of ethene (Peitz *et al.*, 2010). Here we describe the reaction of this ligand with iodomethane. Surprisingly, it is not the expected phosphonium salt with an intact PNPN-framework, but rather the title compound which is formed in this reaction, due to a cleavage of one of the PN-bonds. The reason for this unusual reaction course is unclear.

Two phenyl rings in the molecule of the title compound form a dihedral angle of 82.98 (5)° (Fig. 1). All angles around the quarternized phosphorus atom are nearly tetrahedral, with the exception of N1—P1—C4 which is significantly widened [115.25 (7)°]. A weak donor-acceptor interaction N1—H1A···I1 [N1—H1A 0.80 (2), H1A···I1 2.88 (2), N1···I1 3.6641 (14) Å, N1—H1A···I1 165.1 (17)°] is observed in the structure.

### Experimental

0.062 ml (1.0 mmol) of MeI was added to a stirred solution of 286 mg (0.7 mmol) of Ph_2_PN(^i^Pr)P(Ph)N(^i^Pr)H in 25 ml of diethylether and the resulting solution was stored at room temperature for 48 h. Subsequently, the white precipitate was filtered off, washed with n-hexane and recrystallized in a dichloromethane/n-hexane mixture to yield 154 mg (0.4 mmol) of colourless crystals of the title compound, which were suitable for X-ray crystal structure analysis and fully characterized by standard analytical methods *e.g.*^31^P NMR: (CD_2_Cl_2_): 38,1 p.p.m.

### Refinement

H1A which is attached to N1 was found from difference Fourier map and refined isotropically [N1—H1A 0.81 (2) Å]. All other H atoms were placed in idealized positions with d(C—H) = 0.98 (CH_3_) and 0.95–1.00 Å (CH) and refined using a riding model with *U*_iso_(H) fixed at 1.5 *U*_eq_(C) for CH_3_ and 1.2 *U*_eq_(C) for CH.

### Crystal data

**Table:**

C_16_H_21_NP^+^·I^−^	*F*(000) = 768
*M**_r_* = 385.21	*D*_x_ = 1.517 Mg m^−^^3^
Monoclinic, *P*2_1_/*n*	Mo *K*α radiation, λ = 0.71073 Å
Hall symbol: -P 2yn	Cell parameters from 9114 reflections
*a* = 9.0283 (2) Å	θ = 2.0–28.4°
*b* = 20.2810 (6) Å	µ = 1.98 mm^−^^1^
*c* = 9.2298 (3) Å	*T* = 150 K
β = 93.492 (2)°	Prism, colourless
*V* = 1686.87 (8) Å^3^	0.45 × 0.30 × 0.16 mm
*Z* = 4	

### Data collection

**Table:**

Stoe IPDS II diffractometer	4035 independent reflections
Radiation source: fine-focus sealed tube	3604 reflections with *I* > 2σ(*I*)
graphite	*R*_int_ = 0.024
ω scans	θ_max_ = 27.9°, θ_min_ = 2.0°
Absorption correction: numerical (*X-SHAPE* and *X-RED32*; Stoe & Cie, 2005)	*h* = −11→11
*T*_min_ = 0.484, *T*_max_ = 0.741	*k* = −26→26
28937 measured reflections	*l* = −12→12

### Refinement

**Table:**

Refinement on *F*^2^	Primary atom site location: structure-invariant direct methods
Least-squares matrix: full	Secondary atom site location: difference Fourier map
*R*[*F*^2^ > 2σ(*F*^2^)] = 0.019	Hydrogen site location: inferred from neighbouring sites
*wR*(*F*^2^) = 0.048	H atoms treated by a mixture of independent and constrained refinement
*S* = 1.05	*w* = 1/[σ^2^(*F*_o_^2^) + (0.0324*P*)^2^ + 0.1321*P*] where *P* = (*F*_o_^2^ + 2*F*_c_^2^)/3
4035 reflections	(Δ/σ)_max_ = 0.001
179 parameters	Δρ_max_ = 0.63 e Å^−^^3^
0 restraints	Δρ_min_ = −0.43 e Å^−^^3^

### Special details

**Table:**

Geometry. All e.s.d.'s (except the e.s.d. in the dihedral angle between two l.s. planes) are estimated using the full covariance matrix. The cell e.s.d.'s are taken into account individually in the estimation of e.s.d.'s in distances, angles and torsion angles; correlations between e.s.d.'s in cell parameters are only used when they are defined by crystal symmetry. An approximate (isotropic) treatment of cell e.s.d.'s is used for estimating e.s.d.'s involving l.s. planes.
Refinement. Refinement of *F*^2^ against ALL reflections. The weighted *R*-factor *wR* and goodness of fit *S* are based on *F*^2^, conventional *R*-factors *R* are based on *F*, with *F* set to zero for negative *F*^2^. The threshold expression of *F*^2^ > σ(*F*^2^) is used only for calculating *R*-factors(gt) *etc*. and is not relevant to the choice of reflections for refinement. *R*-factors based on *F*^2^ are statistically about twice as large as those based on *F*, and *R*- factors based on ALL data will be even larger.

### Fractional atomic coordinates and isotropic or equivalent isotropic displacement parameters (Å^2^)

**Table:**

	*x*	*y*	*z*	*U*_iso_*/*U*_eq_	
C1	0.3442 (2)	0.76211 (8)	0.30748 (19)	0.0309 (3)	
H1B	0.4488	0.7578	0.2787	0.037*	
C2	0.3472 (3)	0.78356 (12)	0.4653 (2)	0.0532 (6)	
H2A	0.3970	0.8264	0.4762	0.080*	
H2B	0.4011	0.7508	0.5260	0.080*	
H2C	0.2454	0.7874	0.4955	0.080*	
C3	0.2667 (2)	0.69656 (9)	0.2829 (2)	0.0404 (4)	
H3A	0.1616	0.7012	0.3019	0.061*	
H3B	0.3127	0.6635	0.3487	0.061*	
H3C	0.2755	0.6825	0.1822	0.061*	
C4	0.36979 (17)	0.94492 (8)	0.24664 (16)	0.0237 (3)	
C5	0.4810 (2)	0.99058 (9)	0.22218 (18)	0.0315 (3)	
H5	0.5504	0.9820	0.1511	0.038*	
C6	0.4901 (2)	1.04863 (9)	0.3019 (2)	0.0355 (4)	
H6	0.5654	1.0800	0.2853	0.043*	
C7	0.3891 (2)	1.06068 (9)	0.40561 (19)	0.0328 (4)	
H7	0.3950	1.1006	0.4596	0.039*	
C8	0.28023 (19)	1.01536 (9)	0.43122 (18)	0.0306 (3)	
H8	0.2124	1.0239	0.5037	0.037*	
C9	0.26912 (18)	0.95730 (8)	0.35172 (16)	0.0267 (3)	
H9	0.1934	0.9262	0.3689	0.032*	
C10	0.23107 (17)	0.89557 (8)	−0.02177 (16)	0.0236 (3)	
C11	0.1988 (2)	0.84705 (9)	−0.12705 (18)	0.0297 (3)	
H11	0.2304	0.8029	−0.1107	0.036*	
C12	0.1206 (2)	0.86407 (10)	−0.25494 (18)	0.0344 (4)	
H12	0.0985	0.8315	−0.3270	0.041*	
C13	0.0743 (2)	0.92834 (11)	−0.27819 (19)	0.0368 (4)	
H13	0.0212	0.9397	−0.3667	0.044*	
C14	0.1045 (2)	0.97639 (10)	−0.17426 (19)	0.0359 (4)	
H14	0.0716	1.0204	−0.1909	0.043*	
C15	0.18339 (18)	0.95991 (9)	−0.04522 (17)	0.0284 (3)	
H15	0.2046	0.9926	0.0267	0.034*	
N1	0.26667 (16)	0.81103 (7)	0.21097 (14)	0.0255 (3)	
P1	0.34534 (4)	0.87284 (2)	0.13541 (4)	0.02117 (8)	
I1	−0.103895 (11)	0.834024 (5)	0.344262 (11)	0.02825 (4)	
C16	0.52126 (19)	0.84672 (9)	0.07819 (19)	0.0300 (3)	
H16A	0.5904	0.8409	0.1633	0.045*	
H16B	0.5603	0.8801	0.0139	0.045*	
H16C	0.5097	0.8048	0.0260	0.045*	
H1A	0.180 (2)	0.8158 (10)	0.223 (2)	0.025 (5)*	

### Atomic displacement parameters (Å^2^)

**Table:**

	*U*^11^	*U*^22^	*U*^33^	*U*^12^	*U*^13^	*U*^23^
C1	0.0332 (9)	0.0258 (8)	0.0339 (8)	0.0029 (7)	0.0046 (7)	0.0075 (6)
C2	0.0787 (17)	0.0453 (12)	0.0337 (10)	−0.0003 (11)	−0.0116 (10)	0.0064 (9)
C3	0.0504 (12)	0.0264 (9)	0.0463 (10)	−0.0014 (8)	0.0172 (9)	0.0015 (8)
C4	0.0234 (7)	0.0247 (8)	0.0226 (6)	−0.0009 (6)	−0.0008 (5)	0.0017 (6)
C5	0.0309 (8)	0.0349 (9)	0.0286 (7)	−0.0084 (7)	0.0017 (6)	0.0007 (7)
C6	0.0379 (10)	0.0307 (9)	0.0371 (9)	−0.0119 (7)	−0.0042 (7)	0.0037 (7)
C7	0.0384 (10)	0.0247 (8)	0.0339 (8)	0.0015 (7)	−0.0104 (7)	−0.0020 (7)
C8	0.0289 (8)	0.0313 (9)	0.0311 (8)	0.0052 (6)	−0.0021 (6)	−0.0035 (6)
C9	0.0253 (8)	0.0273 (8)	0.0276 (7)	−0.0003 (6)	0.0018 (6)	−0.0009 (6)
C10	0.0201 (7)	0.0284 (8)	0.0224 (7)	−0.0004 (6)	0.0027 (5)	0.0018 (6)
C11	0.0298 (8)	0.0302 (8)	0.0290 (8)	−0.0023 (6)	0.0026 (6)	−0.0022 (6)
C12	0.0296 (9)	0.0466 (11)	0.0269 (8)	−0.0069 (8)	0.0019 (6)	−0.0039 (7)
C13	0.0275 (9)	0.0555 (12)	0.0272 (8)	−0.0006 (8)	−0.0007 (6)	0.0089 (7)
C14	0.0314 (9)	0.0403 (10)	0.0360 (9)	0.0068 (7)	0.0016 (7)	0.0105 (7)
C15	0.0268 (8)	0.0301 (8)	0.0285 (7)	0.0010 (6)	0.0031 (6)	0.0017 (6)
N1	0.0217 (7)	0.0265 (7)	0.0287 (6)	−0.0002 (5)	0.0044 (5)	0.0031 (5)
P1	0.01969 (18)	0.02299 (19)	0.02100 (16)	0.00007 (14)	0.00262 (13)	0.00071 (13)
I1	0.02589 (6)	0.02866 (7)	0.03035 (6)	−0.00158 (4)	0.00287 (4)	−0.00291 (4)
C16	0.0237 (8)	0.0349 (9)	0.0319 (8)	0.0047 (6)	0.0067 (6)	0.0020 (7)

### Geometric parameters (Å, °)

**Table:**

C1—N1	1.481 (2)	C8—H8	0.9500
C1—C3	1.513 (3)	C9—H9	0.9500
C1—C2	1.519 (3)	C10—C15	1.387 (2)
C1—H1B	1.0000	C10—C11	1.401 (2)
C2—H2A	0.9800	C10—P1	1.7886 (15)
C2—H2B	0.9800	C11—C12	1.382 (2)
C2—H2C	0.9800	C11—H11	0.9500
C3—H3A	0.9800	C12—C13	1.381 (3)
C3—H3B	0.9800	C12—H12	0.9500
C3—H3C	0.9800	C13—C14	1.383 (3)
C4—C9	1.392 (2)	C13—H13	0.9500
C4—C5	1.394 (2)	C14—C15	1.391 (2)
C4—P1	1.7922 (16)	C14—H14	0.9500
C5—C6	1.388 (3)	C15—H15	0.9500
C5—H5	0.9500	N1—P1	1.6195 (15)
C6—C7	1.383 (3)	N1—H1A	0.81 (2)
C6—H6	0.9500	P1—C16	1.7845 (17)
C7—C8	1.377 (3)	C16—H16A	0.9800
C7—H7	0.9500	C16—H16B	0.9800
C8—C9	1.388 (2)	C16—H16C	0.9800
			
N1—C1—C3	107.52 (15)	C4—C9—H9	120.3
N1—C1—C2	111.42 (15)	C15—C10—C11	120.21 (15)
C3—C1—C2	112.10 (16)	C15—C10—P1	121.82 (12)
N1—C1—H1B	108.6	C11—C10—P1	117.82 (13)
C3—C1—H1B	108.6	C12—C11—C10	119.39 (17)
C2—C1—H1B	108.6	C12—C11—H11	120.3
C1—C2—H2A	109.5	C10—C11—H11	120.3
C1—C2—H2B	109.5	C13—C12—C11	120.15 (17)
H2A—C2—H2B	109.5	C13—C12—H12	119.9
C1—C2—H2C	109.5	C11—C12—H12	119.9
H2A—C2—H2C	109.5	C12—C13—C14	120.82 (16)
H2B—C2—H2C	109.5	C12—C13—H13	119.6
C1—C3—H3A	109.5	C14—C13—H13	119.6
C1—C3—H3B	109.5	C13—C14—C15	119.58 (18)
H3A—C3—H3B	109.5	C13—C14—H14	120.2
C1—C3—H3C	109.5	C15—C14—H14	120.2
H3A—C3—H3C	109.5	C10—C15—C14	119.85 (16)
H3B—C3—H3C	109.5	C10—C15—H15	120.1
C9—C4—C5	120.01 (15)	C14—C15—H15	120.1
C9—C4—P1	118.91 (12)	C1—N1—P1	125.03 (12)
C5—C4—P1	120.91 (13)	C1—N1—H1A	115.3 (14)
C6—C5—C4	119.83 (17)	P1—N1—H1A	115.0 (14)
C6—C5—H5	120.1	N1—P1—C16	108.61 (8)
C4—C5—H5	120.1	N1—P1—C10	107.60 (7)
C7—C6—C5	119.77 (17)	C16—P1—C10	108.44 (8)
C7—C6—H6	120.1	N1—P1—C4	115.25 (7)
C5—C6—H6	120.1	C16—P1—C4	109.32 (8)
C8—C7—C6	120.56 (16)	C10—P1—C4	107.41 (7)
C8—C7—H7	119.7	P1—C16—H16A	109.5
C6—C7—H7	119.7	P1—C16—H16B	109.5
C7—C8—C9	120.33 (17)	H16A—C16—H16B	109.5
C7—C8—H8	119.8	P1—C16—H16C	109.5
C9—C8—H8	119.8	H16A—C16—H16C	109.5
C8—C9—C4	119.49 (16)	H16B—C16—H16C	109.5
C8—C9—H9	120.3		

### Hydrogen-bond geometry (Å, °)

**Table:**

*D*—H···*A*	*D*—H	H···*A*	*D*···*A*	*D*—H···*A*
N1—H1A···I1	0.81 (2)	2.88 (2)	3.6641 (15)	164.9 (18)
